# Supplementary material for: Surface Microbiome Profiling of Dental Elevators Using Third‐Generation Sequencing: Implications for Infection Control in Dental Practice
Source: Microbiologyopen. 2025 Dec 8;14(6):e70178. doi: 10.1002/mbo3.70178 (PMC12685768; doi:10.1002/mbo3.70178)
Supplement: Supplementary file 1 — customer_backup. [file MBO3-14-e70178-s001.zip › customer_backup/customer_backup/taxa_summary/krona/samples/M05.Krona.html]

Javascript must be enabled to view this page.

members
magnitude
magnitudeUnassigned

M05.krona

57394

57394

2

2

2

2

2

1

1

3149

6

6

6

6

6

126

126

7

7

7

67

67

67

52

6

6

1

1

6

6

22

2

20

17

1

16

218

37

3

1

1

2

2

34

34

34

41

2

2

2

1

1

1

32

32

32

3

3

3

2

2

2

1

1

1

5

5

5

5

135

73

3

3

70

1

69

7

7

1

2

4

35

35

35

20

1

1

18

18

1

1

238

160

46

34

13

3

7

8

3

10

1

9

1

1

1

1

114

2

2

31

31

80

80

1

1

78

51

51

10

39

2

27

2

2

25

25

2555

147

147

147

23

15

101

8

1

1

1

1

1

1

1

1

534

519

57

57

5

5

2

2

8

8

405

18

387

42

42

15

2

1

1

10

5

1

2

2

3

3

4

4

1

1

2

2

1

1

66

19

1

1

18

18

3

3

3

44

34

4

30

10

10

10

10

9

1

1

1

5

1

1

1

8

2

2

2

3

3

3

1

1

1

1

1

1

1

1

1

111

111

111

2

17

71

19

1

1

1672

1259

1205

1205

10

10

2

2

1

1

9

9

32

21

2

6

3

8

2

2

4

1

3

2

2

2

2

2

401

1

1

2

2

395

41

354

3

1

2

2

2

2

1

1

1

1

6

6

6

6

2

3

1

951

951

951

951

951

247

23

65

5

19

14

40

6

110

46

157

109

40

70

227

227

227

227

227

227

1066

1066

1066

1066

47

47

1019

1019

62

62

62

62

20

5

11

4

42

42

1

1

1

1

1

1

33

15

4

4

2

2

1

1

1

1

11

3

3

3

1

1

1

1

1

1

3

2

2

1

1

3

3

3

18

18

1

1

1

3

3

3

2

2

2

6

6

6

6

6

6

3

3

3

3

3

2

1

978

21

21

21

21

21

54

54

54

29

29

20

8

12

5

5

903

903

60

1

1

59

59

843

843

843

156

156

156

13

13

13

3

3

3

125

2

2

44

44

8

8

71

71

14

14

14

1

1

1

45

25

5

5

5

5

20

2

2

1

1

18

2

2

4

4

12

12

12

10

10

10

2

6

2

2

2

1

1

1

1

1

1

1

1

1

1

1

1

1

1

6

6

1

1

1

1

1

1

4

4

4

2

2

2

2

2

2

1

1

1

1

1

1

30

1

1

1

1

1

2

2

2

1

1

1

1

27

27

27

1

1

26

21

5

2070

2070

2070

2070

2070

477

1387

206

39

7

7

7

7

7

32

15

12

10

1

8

1

2

2

3

3

3

16

16

16

4

12

1

1

1

1

6

4

4

2

2

2

1

1

1

1

1

1

2

2

2

2

2

14447

925

43

43

43

43

15

1

1

1

11

1

1

1

1

9

9

3

3

3

18

18

12

12

6

6

12

12

1

1

10

10

1

1

4

4

4

4

1

1

1

1

92

92

1

1

5

1

2

1

1

2

2

2

1

1

1

1

20

17

1

2

4

2

2

57

3

1

1

11

21

15

1

4

16

16

16

16

56

56

1

1

53

53

1

1

1

1

519

12

4

4

1

1

7

2

5

2

2

2

41

5

1

1

3

36

36

1

1

1

7

1

1

6

1

4

1

3

3

3

5

3

3

2

2

55

2

2

2

2

25

25

2

1

1

24

1

23

8

2

2

4

4

2

2

284

1

1

78

74

4

1

1

24

2

10

10

2

84

84

96

1

91

1

3

4

4

4

97

9

9

2

2

6

6

1

1

24

2

2

4

6

10

55

55

2

2

2

2

58

1

1

1

57

11

11

2

1

1

1

1

2

2

41

41

1

1

1

1

6

1

1

1

4

4

4

1

1

1

82

82

18

18

1

1

43

41

2

2

2

1

1

16

5

11

1

1

13522

3

3

3

3

2152

2

1

1

1

1

2

2

2

402

275

275

42

42

14

14

71

71

1474

1

1

34

34

1058

74

984

381

342

39

272

272

272

10800

21

21

21

51

51

51

4

4

4

1

1

1

8

6

6

2

2

2538

1

1

2

2

2397

2397

126

1

112

8

5

12

1

3

1

7

6

1

1

4

1

3

1

1

122

122

122

4861

4861

4861

6

6

6

3178

58

58

1

1

10

10

1

1

1

1

427

1

426

4

1

2

1

11

11

84

84

22

22

2559

2559

4

3

3

1

1

7

5

1

1

3

3

1

1

2

2

2

2

2

2

2

1

1

1

1

1

1

1

1

1

1

1

1

45

8

2

2

1

1

4

4

1

1

37

8

8

4

1

2

1

25

1

1

3

10

1

1

1

2

5

67

67

67

67

1

1

1

1

442

18

18

18

110

110

99

11

39

29

29

10

10

275

119

119

156

18

138

2

2

2

2

1

1

1

1

3

3

3

3

3

1

2

18

6

6

6

1

1

1

1

1

1

3

2

1

12

7

5

1

1

4

2

2

1

1

1

1

1

1

5

5

5

2

3

30877

8235

21

21

1

1

20

19

1

257

1

1

1

256

16

16

240

240

3

3

3

3

14

14

14

2

12

2781

2522

1

1

2

2

115

30

85

77

75

2

9

9

3

2

1

1

1

3

3

40

40

29

29

54

1

26

7

7

1

5

5

1

1

1

1

46

1

45

480

139

7

198

131

5

49

44

5

44

2

41

1

91

19

68

4

1

1

135

135

36

36

1

1

186

186

27

27

10

10

138

12

99

27

1

1

4

4

84

84

5

5

7

6

1

99

28

71

741

7

539

3

50

2

113

9

5

8

5

2

2

2

2

2

257

257

257

4699

2449

1216

1216

455

4

113

338

10

10

758

747

11

9

9

1

1

2069

4

4

1928

1928

106

106

2

2

29

28

1

181

1

1

1

1

179

179

28

28

27

22

2

2

1

1

1

7

2

2

2

3

3

3

2

2

2

63

6

6

6

4

4

4

53

53

53

362

1

1

1

213

6

6

7

1

6

8

8

1

1

36

3

30

3

38

38

2

2

14

1

1

1

6

5

43

43

1

1

32

1

31

1

1

24

24

18

18

18

1

1

1

117

1

1

20

7

3

3

7

43

39

4

45

3

1

1

17

23

6

6

2

1

1

12

11

11

1

1

20908

125

88

7

7

81

2

1

1

3

9

36

5

1

6

15

2

37

16

16

5

1

4

9

9

1

1

6

5

1

8

8

8

8

21

21

1

1

20

20

431

248

181

181

50

37

13

8

6

2

9

9

183

9

8

1

15

15

3

3

24

24

16

16

5

5

55

55

9

9

2

2

28

5

23

7

7

4

4

6

6

494

171

1

1

170

1

169

323

323

323

7

7

7

1

1

1

2

1

1

1

1

1

1

1730

1730

1730

1729

1

7

6

6

6

1

1

1

18083

9

9

6

3

553

553

553

14852

14846

19

2069

658

9825

1266

19

849

1

139

1

6

6

6

6

6

2590

52

52

10

7

2

1

524

250

6

98

170

497

246

62

1

157

31

16

16

1463

1369

5

89

21

21

1

1

5

5

1

1

73

72

72

1

1

1

1

1

1

1

1

1

1

1

1733

1733

155

3

3

152

114

38

1574

1088

1088

486

2

1

482

1

4

4

4

39

38

5

5

5

5

23

23

23

23

10

10

10

10

1

1

1

1

1

7

7

7

7

7

7

9

8

8

8

8

8

1

1

1

1

1

3165

3164

76

76

3

1

2

5

5

1

1

1

1

56

1

7

48

2

1

1

1

1

2

2

2

2

3

3

101

72

3

3

5

5

61

40

21

2

2

1

1

14

14

14

15

15

15

2915

195

1

1

48

48

146

146

203

137

137

66

3

1

13

3

29

12

5

7

7

7

31

31

31

36

36

7

29

589

589

57

81

451

177

177

4

1

1

135

1

1

2

13

2

1

16

797

13

13

733

716

17

51

1

23

6

5

16

588

7

1

6

1

1

2

2

319

22

47

79

171

259

13

19

81

13

133

292

292

292

1

1

1

1

71

1

1

1

57

1

1

56

56

3

3

3

3

2

2

1

1

7

1

1

6

1

2

1

1

1

1

1

1

1

1

6

6

6

1

1

1

5

5

5
